# Supplementary material for: All-at-once RNA folding with 3D motif prediction framed by evolutionary information
Source: Nat Methods. 2025 Oct 3;22(10):2094–106. doi: 10.1038/s41592-025-02833-w (PMC12510878; doi:10.1038/s41592-025-02833-w)
Supplement: Supplementary file 3 — Source code to run CaCoFold-R3D. Data and results. [file 41592_2025_2833_MOESM3_ESM.gz › supplemental_material/results/CaCoFold-R3D/CaCoFold-R3D_Rfam_vshuffle/RF02202_UCA1.R2R.sto.pdf]

RF02202_UCA1

5′ - A G G C C G A R A G C C G A U C A G A C A A A C A A C C U A C A A C C C U U A A G C U C C U G R C A G Y G C C ● A G Y C A A G G C Y A U G C U U C C A
